# Development of An Impedimetric Aptasensor for the Detection of *Staphylococcus aureus*

**DOI:** 10.3390/ijms18112484

**Published:** 2017-11-21

**Authors:** Peggy Reich, Regina Stoltenburg, Beate Strehlitz, Dieter Frense, Dieter Beckmann

**Affiliations:** 1 Institut für Bioprozess- und Analysenmesstechnik e.V., 37308 Heilbad Heiligenstadt, Germany; Dieter.Frense@iba-heiligenstadt.de (D.F.); Dieter.Beckmann@iba-heiligenstadt.de (D.B.); 2 UFZ – Helmholtz Centre for Environmental Research, 06120 Halle, Germany; regina.stoltenburg@ufz.de; 3 UFZ – Helmholtz Centre for Environmental Research, 04318 Leipzig, Germany; beate.strehlitz@ufz.de

**Keywords:** aptamer, staphylococcal protein A, label-free, biosensing techniques, rapid detection, self-assembly, limit of detection, protein binding, ferri-/ferrocyanide, gold electrode

## Abstract

In combination with electrochemical impedance spectroscopy, aptamer-based biosensors are a powerful tool for fast analytical devices. Herein, we present an impedimetric aptasensor for the detection of the human pathogen *Staphylococcus aureus*. The used aptamer targets protein A, a surface bound virulence factor of *S. aureus*. The thiol-modified protein A-binding aptamer was co-immobilized with 6-mercapto-1-hexanol onto gold electrodes by self-assembly. Optimization of the ratio of aptamer to 6-mercapto-1-hexanol resulted in an average density of 1.01 ± 0.44 × 10^13^ aptamer molecules per cm^2^. As shown with quartz crystal microbalance experiments, the immobilized aptamer retained its functionality to bind recombinant protein A. Our impedimetric biosensor is based on the principle that binding of target molecules to the immobilized aptamer decreases the electron transfer between electrode and ferri-/ferrocyanide in solution, which is measured as an increase of impedance. Microscale thermophoresis measurements showed that addition of the redox probe ferri-/ferrocyanide has no influence on the binding of aptamer and its target. We demonstrated that upon incubation with various concentrations of *S. aureus*, the charge-transfer resistance increased proportionally. The developed biosensor showed a limit of detection of 10 CFU·mL^−1^ and results were available within 10 minutes. The biosensor is highly selective, distinguishing non-target bacteria such as *Escherichia coli* and *Staphylococcus epidermidis*. This work highlights the immense potential of impedimetric aptasensors for future biosensing applications.

## 1. Introduction

*Staphylococcus aureus* is a major pathogen for humans. It is a common cause of infections, from minor ones such as abscesses and sinusitis to life-threatening diseases such as bacteremia, endocarditis and sepsis [1]. Its antibiotic-resistant strains, e.g., methicillin-resistant *S. aureus* (MRSA) are a serious problem in healthcare [2]. Besides being the origin of hospital-acquired infections, *S. aureus* produces seven different toxins that cause food poisoning [3,4,5]. It is generally accepted, that 10^5^ cells per g of food produce sufficient enterotoxins to cause food poisoning [6].

Since the relevance of this pathogen was discovered, many approaches in the development of rapid detection methods for infection control were investigated, as reviewed by Law et al. [7] and Zhao et al. [8]. According to these reviews, traditional methods, such as plate counts using selective agar, convince with their simplicity, low costs and high accuracy but take 4 to 6 days to yield results. Nevertheless, they are still regarded as the gold standard. One promising alternative method is polymerase chain reaction (PCR). The commercially available Xpert MRSA assay (Cepheid International, Sunnyvale, CA, USA) for example requires 2 h from DNA extraction to assay result [9]. However, complex sample preparation by trained staff is needed.

According to Zhao et al., the most rapid detection methods are based on biosensor technology. Biosensors are devices, which use biological components as recognition elements to provide specific affinity to the desired target. The recognition element is coupled to a transducer, which transforms the biological into an electrical signal [10]. To be commercially successful, a biosensor has to meet several requirements, e.g., low cost, fast response and high sensitivity. Therefore, despite its complexity, many researchers recognize the high potential of electrochemical impedance spectroscopy (EIS).

EIS is a fast label-free technique to measure the properties of electrode surfaces and bulk electrolytes. Owed to the progress in engineering and electronics during the last decades, high performance miniaturized impedance instruments are available for a relatively low budget [11]. EIS was used successfully for biosensors with various recognition elements [12,13]. For example, Bekir et al., developed an electrochemical immunosensor using antibodies against *S. aureus* [14]. They report a detection limit of 10 CFU·mL^−1^ of *S. aureus*, exploiting the impedance change of the electrode surface caused by the affinity reaction of the immobilized antibodies.

To overcome the limitations of antibodies, such as high manufacturing costs, instability to high temperatures and short shelf life, aptasensors employ aptamers as recognition element [15]. Aptamers are synthetic, single-stranded nucleic acid molecules that can fold into complex three-dimensional structures allowing them to bind targets based on structure recognition with high affinity and specificity. They are selected using the SELEX procedure (systematic evolution of ligands by exponential enrichment), an iterative in vitro selection and amplification method [16].

Electrochemical aptasensors were reviewed by Willner et al. [17]: besides the well-known thrombin aptamer [18], other impedimetric aptasensors emerged ranging from the detection of potassium ions [19] and small molecules, such as ethanolamine [20], to whole cells, e.g., *Salmonella typhimurium* [21]. Shahdordizadeh et al., provided a review of recent advances in optical and electrochemical aptasensors for the detection of *S. aureus* [22]. They report on aptamers selected against staphylococcal toxins, staphylococcal teichoic acid, staphylococcal protein A and *S. aureus* as whole bacteria. The indirect detection of *S. aureus* via aptamers targeting the toxins excreted by the pathogen are limited due to the difficulty in correlation of the sensor signal to the presence of viable microorganisms. Therefore, direct detection is favored. In the field of optical aptasensors, fluorescence is most prominent, but also one colorimetric aptasensor was developed [23]. Using dielectrophoretic enrichment and fluorescent nanoparticles, Shangguan and coworkers developed an optical aptasensor with a limit of detection (LoD) of 93 CFU·mL^−1^ and an assay time of 2 h [24]. By the use of upconversion nanoparticles, the fluorescence intensity was increased and Duan et al., gained a LoD of 8 CFU·mL^−1^ [25]. Chang et al., developed an optical aptasensor for the single cell detection of *S. aureus* within 1.5 h [26]. The detection principle is based on resonance light scattering of modified gold nanoparticles. Optical sensors have the disadvantage that complex biological samples often interfere with the detection process. Furthermore, electrochemical methods are appreciated for their fast response time, higher sensitivity, low-cost fabrication, simple automation and lower sample volumes. In their review, Shahdordizadeh et al., described five electrochemical aptasensors for the detection of *S. aureus* [22]: Two are based on potentiometry with LoDs of 800 CFU·mL^−1^ [27] and single cell detection [28]. Another used voltammetry to reach a LoD of 1 CFU·mL^−1^ [29] and Lian et al., combined interdigital electrodes (IDE) with quartz crystal sensor to detect the bacteria as low as 12 CFU·mL^−1^ [30]. Jia et al., used a glassy carbon electrode with aptamer modified gold nanoparticles to impedimetric detect a lower limit of 10 CFU·mL^−1^ within 60 min [31].

All mentioned optical and electrochemical aptasensors used different aptamers, but have in common, that the aptamers were selected in a Cell-SELEX, wherein whole cells were used as target for aptamer generation. Although purposive, this has the disadvantage that it stays unknown, which part of the cell surface is targeted by the aptamer. Thus, it is also unknown, which *S. aureus* strains can be bound by these aptamers. *S. aureus* is known for its ability to adapt its genetics quickly to new environments. Nevertheless, the conserved sequence of the immune-evasive factor protein A shows only one mutation in 70 months [32]. The surface bound protein A enhances *S. aureus*’ adhesion to wounds by binding to the von Willebrand factor (vWF) and prevents phagocytosis by binding to the Fc region of various immunoglobulins [33]. Protein A is bound to peptidoglycans on the cell wall of *S. aureus* and not found on other bacteria. Therefore, protein A is an excellent target for the detection of *S. aureus* cells. Also in PCR methods, the *spA* gene, encoding protein A, is used to distinguish between *S. aureus* and other bacteria.

A DNA aptamer targeting staphylococcal protein A was selected by the FluMag-SELEX procedure in 2015 [34,35]. This aptamer development aimed to detect intact bacterial cells of *S. aureus* via the protein A bound to its cell surface. Binding characteristics of the aptamer to protein A were studied intensively by different methods such as bead-based fluorescent binding assay, surface plasmon resonance (SPR), microscale thermophoresis (MST), and enzyme-linked oligonucleotide assay (ELONA) [35,36].

The structural features of an aptamer play a major role in biosensor development. In case of the protein A-binding aptamer, a combination of two structural elements is important for its functionality: First, an intact and free 5′-end, folding into an imperfect stem-loop motif, is crucial for binding to protein A. Second, the aptamer folds into a parallel G-quadruplex structure as demonstrated by circular dichroism spectroscopy [36].

**Figure 1:**
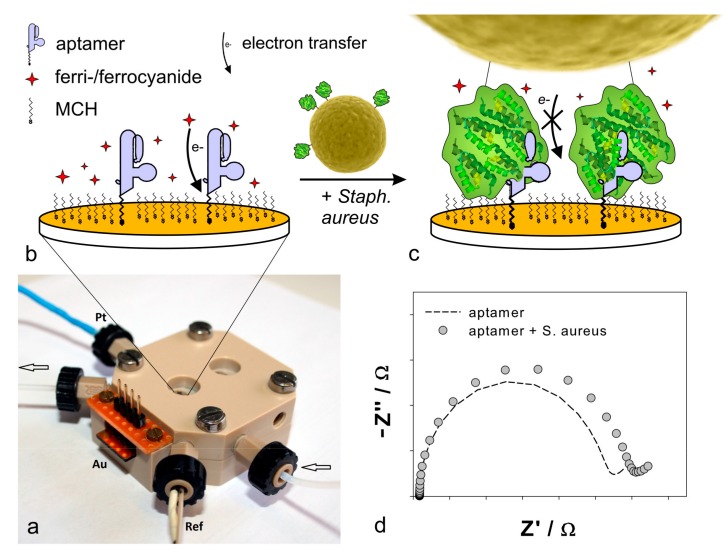
Flow-through measurement chamber and measurement principle. (**a**) Flow-through measurement chamber with liquid inlet and outlet (arrows) and a 3-electrode setup including a golden working electrode (Au), a platinum counter electrode (Pt) and a reference electrode (Ref); (**b**) Working electrode modified with aptamer (violet) and 6-mercapto-1-hexanol (MCH) in a buffer containing the redox probe ferri-/ferrocyanide (red stars); (**c**) Upon binding of protein A (green) on the surface of *S. aureus* (yellow) to the immobilized aptamer (violet), the impedance increased due to the hindrance of electron transfer (curved arrow) between ferri-/ferrocyanide (red stars) in solution and gold electrode surface (orange); (**d**) Characteristic Nyquist-plot of impedance spectra before and after incubation with *S. aureus* (based on data from measurements); Z’: real part of impedance and Z’’: imaginary part of impedance.

In the present study, we developed a biosensor detecting *Staphylococcus aureus* by its surface bound protein A, which is highly conserved and only found on *S. aureus*. The protein A-binding aptamer served as biological recognition element. In combination with electrochemical impedance spectroscopy as measurement method, rapid and label-free detection was achieved. By immobilization of thiol-modified aptamer on gold electrodes by self-assembly, binding of *S. aureus* was detected in a flow-through chamber with a three-electrode setup in buffer solution containing ferri-/ferrocyanide. Upon binding of *S. aureus*, the impedance increased due to the hindrance of the electron transfer between ferri-/ferrocyanide and the electrode surface (Figure 1). Herein, we elucidate the development of an impedimetric aptasensor and present novel insights on the use of aptamer-based electrochemical biosensors for the rapid and selective detection of *S. aureus*.

## 2. Results and Discussion

### 2.1. Functionalization of the Gold Electrodes

**Figure 2:**
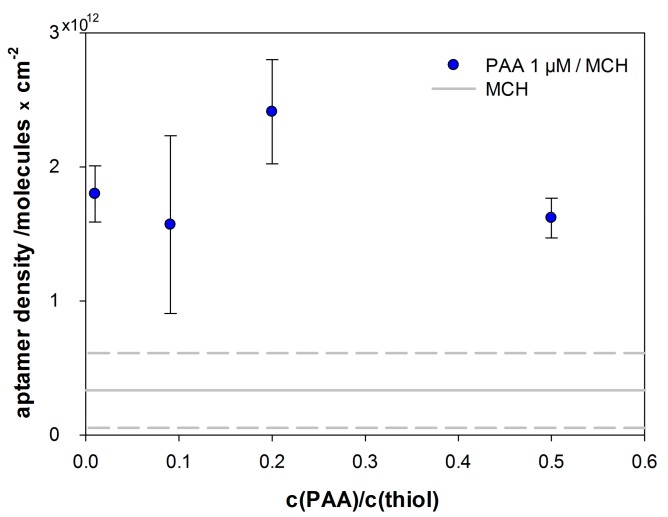
Aptamer density on the electrode surface depending on the protein A-binding aptamer (PAA)/ 6-mercapto-1-hexanol (MCH) molar ratio upon immobilization based on four experiments for each ratio; the grey lines show the average density and standard deviation determined for a MCH monolayer.

Affinity of the protein A-binding aptamer (PAA) to its target has been intensively studied by Stoltenburg et al., using SPR-based measurements with the Biacore X100 [35]. They applied both, the protein A and PAA, respectively as biotinylated receptor, which was immobilized on a streptavidin-coated sensor surface. In the development of the impedimetric sensor, we modified the aptamer with C6-Spacer and thiol for immobilization via self-assembly. To enable high densities of the protein A-binding aptamer (PAA) on the surface, we used the co-immobilization strategy, described by Keighley et al. [37]. They found that in the presence of 6-mercapto-1-hexanol (MCH), oligonucleotides stand upright on the surface rather than lying down, thus, occupy less space and allow a higher density. For optimization studies of our sensor surface, we investigated the influence of different ratios of aptamer to total thiol (PAA + MCH) using chronocoulometry as described by Steel et al. [38]. They stated, that the reduction of hexaammineruthenium (III) chloride (RuHex), measured by chronocoulometry, is proportional to the number of oligonucleotides on the surface. Figure 2 shows the results of chronocoulometry measurements on co-immobilized PAA modified gold electrodes in 40 mM Tris buffer containing 200 µM RuHex. The highest density of PAA (2.41 ± 0.39 × 10^12^ PAA/cm^2^) was reached with a ratio of 1:5 (1 µM PAA and 4 µM MCH), thus, this ratio was used for further experiments.

**Figure 3:**
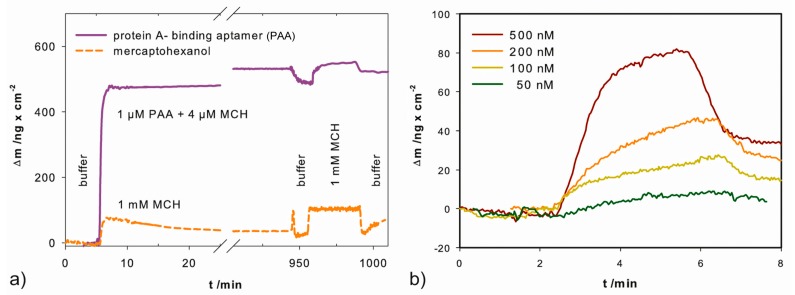
Quartz crystal microbalance measurements: (**a**) mass change Δ*m* versus time of gold covered quartz crystals incubated with aptamer/6-mercapto-1-hexanol (MCH) (violet solid line) or MCH (orange dashed line) and subsequently blocked with 1 mM MCH; (**b**) Δ*m* versus time of an aptamer-modified crystal incubated with different concentrations of protein A.

To ensure successful immobilization and aptamer functionality, we performed analysis using a quartz crystal microbalance as described in Section 3.3. In Figure 3a is shown the mass increase upon PAA immobilization, calculated accordingly from the measured frequency change. The formation of a self-assembled monolayer comprises two steps. The first is the initial attachment, which takes a few seconds, seen in the significant mass increase immediately after introduction of aptamer-solution in Figure 3a. The second step is the arrangement to an ordered monolayer, which takes more than 8 h [39]. Hence, to ensure an ordered monolayer, the electrode was incubated for at least 15 h. As seen in Figure 3a, upon PAA immobilization the mass increased by 500 ng/cm^2^. Backfilling of gaps with MCH increased the mass slightly (~20 ng/cm^2^). In comparison, an electrode covered with a pure MCH monolayer showed a mass increase of only 89 ng/cm^2^. The difference between both provided the mass change due to immobilized PAA. Although immobilized aptamers hamper the formation of an entire layer of MCH, the error is negligible, because the aptamer mass is 142 times higher than the mass of a MCH-molecule. The average mass increase of immobilized PAA was 320 ± 139 ng/cm^2^ (standard deviation obtained from three experiments). This correlates to 1.01 ± 0.44 × 10^13^ aptamers/cm^2^. The aptamer density determined with chronocoulometry (2.41 ± 0.39 × 10^12^ PAA/cm^2^) is 4 times smaller than measured with QCM. This is due to the difference in the measurement techniques and surfaces. In the QCM measurements, not only the mass of the immobilized molecules, but also the adsorbed water and ions as well as the rigidity of the immobilized layer influence the resonance frequency [40]. Also, the different surfaces—a quartz crystal covered with gold and chrome as adhesion layer versus a glass test slide covered with gold and titanium as adhesion layer—contribute to the differences in surface coverage. Although their roughness, <1 nm RMS (= rough mean square) and 376 ± 74 pm RMS respectively, do not diverge significantly. However, both methods confirm successful immobilization and that a high density of aptamers was achieved.

In general, high densities are desired to obtain a higher protein capture capacity resulting in higher sensitivity, but too high densities may lead to steric hindrance preventing correct aptamer folding and binding of target [41]. Assuming an even distribution, the obtained density results in an area of 9.9 × 10^−14^ cm^2^ per aptamer. Assuming an aptamer occupies a squared area, the mean distance between two aptamers is 3.15 nm. Due to dimerization and the formation of quadruplexes [36], the true mean distance of the aptamers is likely >3.15 nm. According to Erickson et al., the partial specific volume of a protein can be calculated from its molecular mass [42]. For the recombinant protein A with a molecular mass of 45 kDa, this volume is 54.54 nm^3^. Assuming protein A has the simplest shape, a sphere, its minimal diameter is 4.71 nm. Therefore, we assumed that the space around an aptamer (>6.3 nm) was sufficient for the binding of protein A.

To prove the functional binding of protein A to the immobilized PAA, we observed the mass change of modified electrodes upon incubation with protein A (Figure 3b). Concentrations of protein A in the range of 100 to 500 nM resulted in signals of 20 to 40 ng/cm^2^, which correlates to 2.68 × 10^11^ and 5.35 × 10^11^ molecules/cm^2^ respectively. Thus, we showed that the immobilized aptamer retained its functionality in binding protein A.

**Figure 4:**
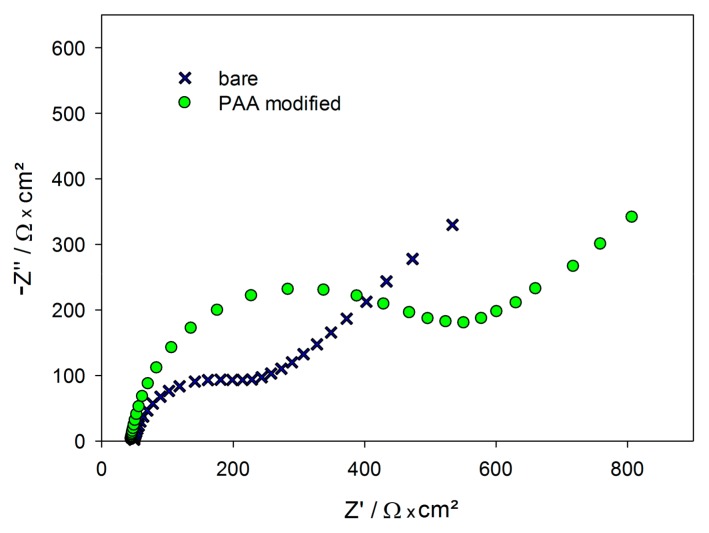
Nyquist plot of the impedance spectra of a blank gold electrode (black crosses) and a PAA/MCH-modified gold electrode (green circles); PAA = protein A-binding aptamer, MCH = 6-mercapto-1-hexanol.

Figure 4 represents the impedance measurements during aptamer immobilization. It shows the Nyquist plots of a blank electrode and an aptamer-modified electrode. The impedance increased significantly after PAA immobilization.

### 2.2. Influence of Ferri-/Ferrocyanide

In faradaic impedimetric measurements, a redox probe for the transfer of electrons from the working electrode to the counter electrode is necessary. The ferri-/ferrocyanide couple is often used due to its fast electron transfer rate (2 × 10^−2^ cm/s) [43]. For biosensor development, it is important to examine, if the redox couple inhibits the affinity of the receptor to its target. To examine the influence of ferri-/ferrocyanide on binding of protein A to PAA, microscale thermophoresis (MST) experiments were performed. MST is a powerful technique to detect biomolecular interaction by quantifying directed movement of molecules along an induced microscopic temperature gradient. It is highly sensitive to changes in hydration shell, charge and size and therefore capable to detect many kinds of biomolecular interactions while both reaction partners remain in solution and no immobilization is required.

In summary, we successfully immobilized PAA via self-assembly with a high density, whereas it retained its functionality of binding protein A.

**Figure 5:**
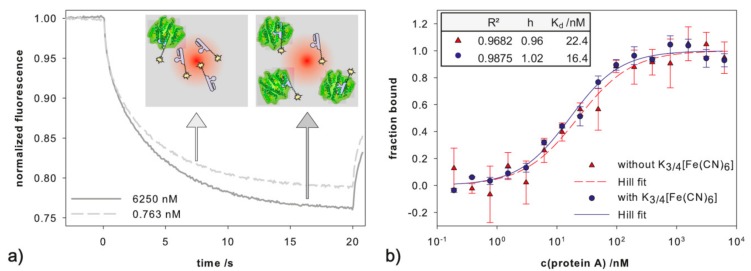
(**a**) Microscale thermophoresis (MST) curves for a low (0.763 nM, dashed line) and a high (6250 nM, solid line) protein A concentration with 38.5 nM labeled PAA (MST power 40%, LED power 100%); (**b**) Binding curves of recombinant protein A with aptamer in absence (red triangles) and presence (blue circles) of 2 mM ferri-/ferrocyanide measured in triplicates—the lines represent the Hill fit.

Figure 5a shows characteristic curves of a MST experiment for a low and a high protein A concentration. The MST fluorescence signal was lower for the high protein A concentration, indicating that in solution, the aptamer-protein A-complex behaved differently than the free aptamer. Binding curves (Figure 5b) in buffer with and without 2 mM ferri-/ferrocyanide were measured as described in Section 3.4. Fitting to the Hill Equation (1) was performed to extract more information from the binding curves:

(1) $$fraction\;bound=\frac{\Delta S}{\Delta S_{\max}}=\;\frac{{\left[T\right]}^{h}}{K_{D}+{\left[T\right]}^{h}}$$

where Δ*S* = signal change, Δ*S_max_* = maximal signal change, *h* = Hill coefficient, *K_D_* = apparent binding constant, [*T*] = target concentration (e.g., protein A).

The determined *K_D_* values in absence and presence of ferri-/ferrocyanide were 22.4 ± 5.8 nM and 16.4 ± 2.5 nM respectively. There is no statistically significant difference between the two curves (paired *t*-test, *p* = 0.255). Thus, we conclude, that ferri-/ferrocyanide has no significant influence on the binding of PAA and protein A.

The *K_D_* value obtained by MST measurements in this work, 22.4 nM, differs from the value reported by Stoltenburg et al., 94.7 nM [35]. To investigate if this difference is due to the labeling- procedure or -site, MST measurements were repeated by 2bind GmbH (Regensburg, Germany). A similar analysis setup was applied using the maleimide-fluorophore on the 5′- and 3′-thiol-tagged PAA. The obtained *K_D_* values were 115.6 nM and 110.8 nM, respectively. Hence, we concluded, that the binding behavior was not affected by the labeling site of the aptamer, for both partners in solution. However, the most distinct difference in the experiments was the concentration of Tween 20 (0.05% used by 2bind GmbH compared to 0.005% used in our first measurement), but the data of both experiments revealed no adhesion to the used capillaries. Thus, the variations in the *K_D_* values in MST measurements are attributed to differences in buffer composition, amplified by the handling of very small volumes (10 µL).

### 2.3. Detection of Protein A by Impedance Spectroscopy

**Figure 6:**
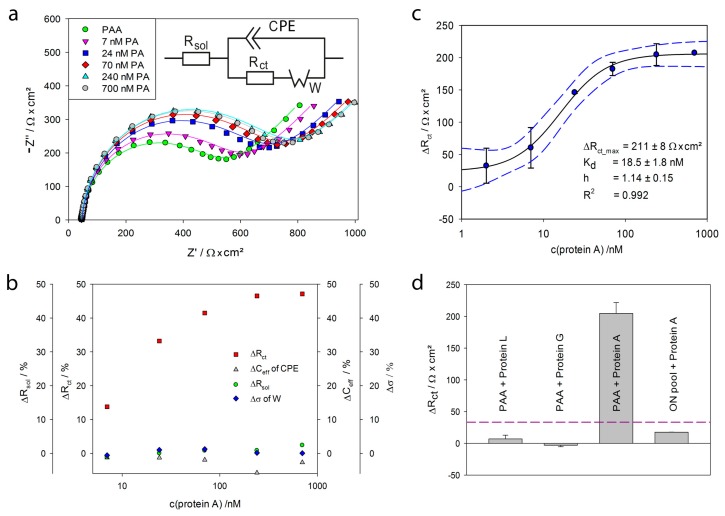
Electrochemical impedance spectroscopy measurements with protein A: (**a**) Nyquist plot of a PAA-modified electrode before (green circles) and after incubation with 7–700 nM protein A measured in FeBB—the fits to the equivalent circuit are shown as lines—modified Randles circuit with *R_sol_*: solution resistance, *CPE*: constant phase element, *R_ct_*: charge-transfer resistance and *W*: Warburg impedance; (**b**) the percentage change of the different fit parameters for all protein A concentrations, *C_eff_*: the effective capacitance was calculated from the *CPE* under the assumption of a parallel distribution of time constants on the electrode surface as described in [44], σ is the parameter for the Warburg element; (**c**) binding curve: Change of the extracted charge transfer resistance Δ*R_ct_* of aptamer-modified electrodes upon incubation of protein A measured in triplicate—dashed blue lines mark the 95% confidence interval; (**d**) unspecific signals: Extracted Δ*R_ct_* of 1 µM protein G, protein L and protein A on PAA-modified electrodes as well as 1 µM protein A on electrodes modified with random oligonucleotides measured in triplicate—the dashed line represents the LoD.

The gold electrodes modified with PAA were mounted in the flow-through chamber (see Figure 1) and exposed to different concentrations of protein A (2–700 nM). Impedance spectra were recorded in FeBB as described in Section 3.5. Every protein A concentration was measured in triplicates, i.e., on three different electrodes. Figure 6a shows the impedance spectra of PAA modified electrodes before and after exposure to 7–700 nM protein A. After incubation with protein A the impedance increased proportionally to the concentration of protein A.

To extract the relevant parameters, the impedance spectra were fitted to the modified Randles circuit (see Figure 6a), wherein *R_sol_* is the solution resistance, *CPE* is the constant phase element for the double layer at the electrode surface, *R_ct_* is the charge-transfer resistance (due to the interaction of ferri-/ferrocyanide with the electrode), and *W* is the Warburg impedance representing the diffusion of ions to the electrode surface. The fitting results are summarized in Table 1. As seen in Figure 6a and Table 1, the fits (lines) show good agreement with the experimental data (markers).

In Figure 6b, the fit parameter changes over all measured protein A concentrations are plotted. The charge transfer resistance *R_ct_* showed a maximum increase of 33%, whereas the other parameters changed less than 4%, verifying that the charge transfer was influenced significantly by the binding of protein A. Thus *R_ct_* was chosen as the significant parameter for further analysis.

The change of the extracted *R_ct_* versus the logarithmic protein A concentration is plotted in Figure 6c. While only a slight increase of *R_ct_* up to 10 nM protein A was observed, an exponential increase in *R_ct_* from 10 nM to 100 nM was measured. Finally, above 100 nM protein A *R_ct_* reached saturation. The resulting sigmoidal curve was fitted to the Hill Equation (2):

(2) $$\Delta R_{ct}=\;\frac{\Delta R_{ct\_max}\;\times \;{\left[T\right]}^{h}}{K_{D}+{\left[T\right]}^{h}}$$

where Δ*R_ct_* = change of the charge transfer resistance, Δ*R_ct_max_* = maximal change, *h* = Hill coefficient, *K_D_* = apparent binding constant, [*T*] = target concentration (e.g., protein A).

Thereby, an apparent *K_D_* value of 18.5 ± 1.8 nM and Hill coefficient *h* of 1.14 ± 0.15 were obtained. The Hill coefficient *h* is slightly higher than 1, indicating a cooperative binding. Stoltenburg et al., already reported avidity effects [35]. They performed competitive experiments with protein A and immunoglobulin, which suggested that the protein A binding site for PAA overlaps with the known binding sites for immunoglobulin [45]. Hence, we can conclude that protein A provides more than one binding site for this aptamer. Reflective Interferometric Fourier Transform Spectroscopy (RIFTS) measurements of protein A binding to PAA, immobilized on porous silicon, resulted in an even higher *h* of 2.61 ± 0.69 [46]. Unlike the herein used planar gold electrodes, the rough and porous silicon surface increases the chance for aptamers being close enough to bind to the same protein A. Both observations substantiate the mentioned avidity affects.

Table 2 summarizes the apparent *K_D_* values obtained with the same aptamer by different detection methods and setups. Affinities were found in the low nanomolar to micromolar range.

Table 2 shows that each of these methods is capable of protein A detection utilizing PAA as receptor in a bioanalytical setup. It suggests that in the same analysis setup by the use of different designs, the measurement range can be adapted for the desired application. As shown in the example in the SPR experiments, immobilization of the aptamer instead of protein A, decreased the *K_D_* almost by 14%. We want to emphasize that the *K_D_* values are strongly dependent on the analysis method and setup used. Therefore, the performance of a biosensor cannot be judged based on the *K_D_* value alone. Every method has to be evaluated for its purpose considering advantages and limitations. i.e., the strengths of EIS as detection method are fast, robust, label-free and non-destructive measurements.

Repeated measurements of PAA-modified electrodes in FeBB resulted in a standard deviation *s* of 11.10 Ω·cm^2^ of the *R_ct_* value. As the limit of detection (LoD) is defined as the lowest target concentration at which the signal is higher than 3·*s*, a LoD of 2.99 ± 0.73 nM was determined using the approximated Hill equation.

Non-specific binding often presents a major challenge in biosensor development. Herein, we investigated the binding of the functionally similar proteins G and L to the aptamer-modified gold electrodes and observed that they neither bind to the surface nor to the aptamer (Figure 6d). Furthermore, the binding of protein A to an electrode, modified with random oligonucleotides, was determined to be 14.8 ± 0.1 Ω·cm^2^ (Figure 4d), which is significantly below LoD (3·*s*).

### 2.4. Detection of Staphylococcus aureus by Impedance Spectroscopy

Besides binding to the defined target protein A, the aptamer was also evaluated for its ability to recognize and bind to intact bacterial cells of *S. aureus*, expressing protein A on the cell surface [36]. Therefore, we performed experiments with our developed impedimetric biosensor and live *S. aureus* cells.

**Figure 7:**
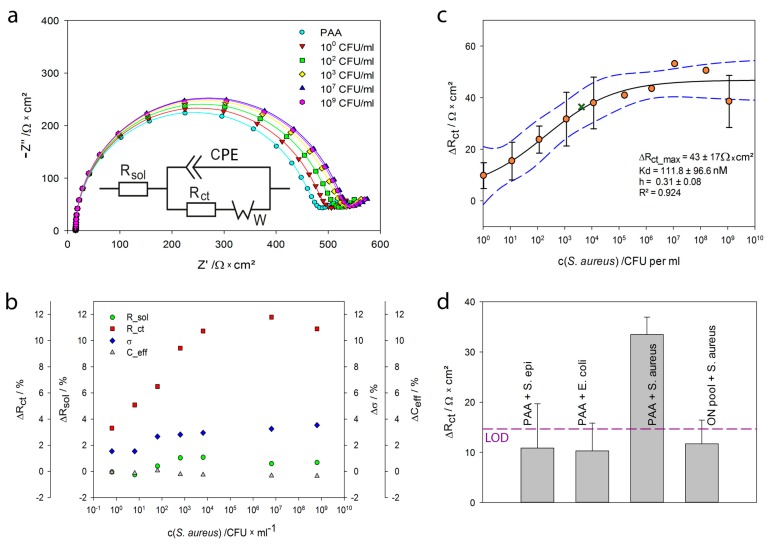
Electrochemical impedance spectroscopy (EIS) with *S. aureus* (**a**) Nyquist plot of a PAA-modified electrode before (cyan circles) and after incubation with 1 to 10^9^ CFU·mL^−1^ measured in FeBB—the fits to the equivalent circuit are shown as lines modified Randles circuit with *R_sol_*: solution resistance, *CPE*: constant phase element, *R_ct_*: charge-transfer resistance and *W*: Warburg impedance; (**b**) percentage change of the different fit parameters for all *S. aureus* concentrations, *C_eff_*: the effective capacitance was calculated from the CPE under the assumption of a parallel distribution of time constants on the electrode surface as described in [45], *σ* is the parameter for the Warburg element; (**c**) binding curve: Change of the extracted charge transfer resistance Δ*R_ct_* of aptamer-modified electrodes upon incubation of 1 to 10^9^ CFU·mL^−1^ *S. aureus* measured in triplicate—dashed blue lines mark the 95% confidence interval, green cross marks a sample of unknown concentration; (**d**) unspecific signals: 10^6^ CFU·mL^−1^ of *Staphylococcus epidermidis*, *Escherichia coli* and *Staphylococcus aureus* on PAA-modified electrodes as well as 10^6^ CFU·mL^−1^ *S. aureus* on electrodes modified with random oligonucleotides measured in triplicate—the dashed line represents the LoD.

The gold electrodes modified with PAA were exposed to different concentrations of *S. aureus* (1 to 10^9^ CFU·mL^−1^). Figure 7a shows impedance spectra of a PAA modified electrode before and after exposure to *S. aureus*, recorded in FeBB. After incubation with *S. aureus*, the impedance increased proportionally to the cell concentration of *S. aureus*. To extract the relevant parameters, the impedance spectra were fitted to the modified Randles circuit (Figure 7a), which describes the phenomena occurring between the electrodes influencing the flow of current. As presented in Figure 7a and Table 3, the fits (lines) show good agreement with the experimental data (markers).

Figure 7b displays the fit parameter change for all measured bacteria concentrations. Only *R_ct_* showed a significant increase of 12%, while the Warburg diffusion increased ~4% and *R_sol_* as well as *C_eff_* changed <2%. This suggests that the bound *S. aureus* influences the electron transfer between electrode and the buffer containing ferri-/ferrocyanide. The *R_ct_* parameter was chosen for further analysis.

In Figure 7c, the change of the extracted *R_ct_* upon incubation with various *S. aureus* concentrations is plotted. Measurements were taken in triplicates, i.e., three electrodes were exposed to each *S. aureus* concentration. The standard deviation of these three measurements is shown as error bars. A diluted sample with 10 *S. aureus* cells per mL resulted in an average *R_ct_* change of 35 Ohm. A sample with 10^5^ cells per mL led to saturation of the sensor surface and the maximum change of 100 Ohm. The fit to the Hill Equation (1) resulted in an apparent *K_D_* value of 111 ± 96 CFU·mL^−1^ and *h* of 0.31 ± 0.08. A measurement with a sample of unknown concentration showed good accordance to the data (green cross). With the approximated Hill equation a bacteria concentration of 5721 ± 2813 CFU·mL^−1^ was determined. Counting the sample under the microscope resulted in a value of 4150 CFU·mL^−1^. The high standard deviation could be minimized by a higher amount of repetitions.

Whereas experiments with protein A resulted in a Hill coefficient slightly higher than 1, indicative for cooperative binding, biosensing experiments with whole cells resulted in a *h* significantly lower than 1, indicating negative cooperativity. This may be due to the size of *S. aureus* cells (~1 µm) and the spacing of protein A molecules on their surface not allowing for several aptamers to bind the same protein A. Another explanation may be the reduced flexibility of the cell surface bound protein A and therefore decreased accessibility of further binding sites for aptamers in the vicinity.

Repeated impedance measurements of a single PAA-modified electrode in FeBB resulted in a standard deviation of *s*(*R_ct_*) = 4.88 Ω·cm^2^. This corresponds to a calculated LoD of 10 CFU·mL^−1^.

A comparison between previously reported *S. aureus* detection assays and the herein demonstrated biosensor is shown in Table 4. In summary, the achieved LoD is comparable to previous reports; however, our biosensor excels in simplicity, automation, low cost and rapid results.

Lastly, we investigated the binding of protein A-deficient bacteria, such as *Staphylococcus epidermidis* and *Escherichia coli* to aptamer-modified gold electrodes and observed no cross-reactivity (Figure 7d). Similar results were found, if *S. aureus* was applied to electrodes modified with random oligonucleotides. Additionally, the binding of *S. aureus* (10^6^ CFU·mL^−1^) to a blank electrode and electrodes modified with a 6-mercapto-1-hexanol (MCH) monolayer, resulted in a negligible average *R_ct_* change of 10.2 ± 2.2 Ω·cm^2^ [49], a value below LoD (3·*s*).

These results are in agreement with results of Stoltenburg et al., reporting that protein A-deficient *S. aureus* strains and gram-negative bacteria, such as *E. coli*, were excluded from aptamer binding [36].

For the practical applicability of our sensor, we recommend that samples shall be extracted into tryptic soy broth or similar media and then diluted 1:100 in BB. The BB contains ions which are required for proper aptamer folding and thus binding to protein A. Simple sample preparation, such as a centrifugation step, showed improved results [49]. The influence of complex sample matrices, such as milk, on the performance of the developed sensor, still has to be examined.

For the food industry, detection of enterotoxins is more crucial than whole cell detection. However, *S. aureus* produces more than 7 different toxins, which all can cause illness. While an existing international standard operation [ISO 19020:2017] describes the screening of staphylococcal enterotoxins SEA, SEB, SECs, SED and SEE, others are excluded (SEG, SHE, SEI, SER, SES and SET). Extraction of the enterotoxins encompasses complex sample preparation, which requires fully equipped food testing laboratories. Furthermore, only enterotoxin assays with low detection limits, such as 0.05 ng/g food, fulfill the requirements [50].

Thus, the predominant approach, especially for small manufacturers, is the detection of *S. aureus* contamination. This approach, based on the Bacteriological Analytical Manual [51], requires culturing on selective agar plates for 2 days. Counting the colony forming units per g food in respect to the processing time is a good indicator for toxin formation. Our sensor could significantly reduce the time required for this approach.

In respect to clinical application, our sensor could improve tests by the rapid identification of bacteria type prior to antibiotic susceptibility testing and thus reduce the costs for isolating patients. Due to the microfluidic setup and the non-destructive impedance measurement, the combination with additional assays and steps will be realizable without great expense.

## 3. Materials and Methods

### 3.1. Reagents

The protein A-binding aptamer PA#2/8 (76 nt) was originally selected by Stoltenburg et al., using the FluMag-SELEX process [34]. A 3′-truncated variant of the full-length aptamer, PA#2/8[S1-58] (58 nt), was applied in the current study: 5′-ATACCAGCTTATTCAATTAGCAACATGAGGGGGAT AGAGGGGGTGGGTTCTCTCGGCT-3′ [35]. This variant is herein referred to as PAA. A pool of randomized oligonucleotides (58 nt) was used as negative control (ON pool). Both were synthesized by Microsynth AG (Balgach, Switzerland), modified with C6-spacer and a thiol at the 3′-end, and purified with polyacrylamide gel electrophoresis.

Recombinant protein A (expressed in *Escherichia coli*, P7837) and 6-mercapto-1-hexanol (MCH, 99%) were purchased from Sigma-Aldrich Chemie GmbH (Taufkirchen, Germany). *Staphylococcus aureus* (DSM 20231), *Staphylococcus epidermidis* (DSM 3269) and *Escherichia coli* (DSM 498) were purchased from Leibniz Institut DSMZ GmbH (Braunschweig, Germany). Potassium- hexacyanoferrate (II) and (III) (K_3/4_[Fe(CN)_6_]) were purchased from Merck Chemicals GmbH (Darmstadt, Germany). Recombinant protein G and protein L (21193 and 21189) were purchased from Pierce Biotechnology (Rockford, USA). Tris-(2-carboxyethyl)-phosphine-hydrochloride (TCEP) was purchased from Carl Roth GmbH + Co. KG (Karlsruhe, Germany). All reagents were of analytical grade and used without further purification. All working solutions were prepared in water purified with a Milli-Q Type-1-system (EMD Millipore Corporation, Billerica, MA, USA; 18 MΩ·cm).

For the aptamer experiments, the binding buffer (BB) as for aptamer selection [35] was used. It consisted of 100 mM NaCl, 20 mM Tris, 10 mM MgCl_2_, 5 mM KCl, 1 mM CaCl_2_ (adjusted with HCl to a pH of 7.6) and was autoclaved, sterile filtered before further use. Protein A was directly dissolved and diluted in BBT, BB containing 0.005% Tween 20, to reduce unspecific binding to surfaces. *S. aureus*, *S. epidermidis* and *E. coli* cells were cultured in tryptic soy broth (TSB) and washed twice in BB. A fraction was dyed with SYTO and counted in a cell chamber of 0.02 mm depth. Washed cell suspensions were diluted to desired concentration in BB. For electrochemical measurements, 2 mM of K_3/4_[Fe(CN)_6_] (equimolar) were added to BB (FeBB).

### 3.2. Preparation of Electrodes

The thiol-modified PAA was preconditioned with TCEP (200 µM/µM thiol) for 20 min to reduce disulfides and heated to 95 °C in BB for 5 min to enable proper folding.

The gold electrodes and gold covered quartz crystals were exposed to ultraviolet light for 5 min and subsequently incubated in hot alkaline piranha solution (5:1:1 water:NH_3_:H_2_O_2_; CAUTION: this acidic mixture reacts violently with organic solvents and must be handled with care!) in an ultrasonic bath for 5 min. After rigorous rinsing with ultrapure water, the electrodes were dried in nitrogen and immediately covered by 1 µM preconditioned PAA and 4 µM MCH in BB followed by incubation at room temperature overnight. Next day, the surface was washed intensively with BB and exposed to 1 mM MCH for 30 min, followed by another wash with BB. The modified electrodes were stored in BB until use.

### 3.3. QCM Measurements

A quartz crystal microbalance (QCM, Q-Sense Analyzer E4, Biolin Scientific Holding AB, Stockholm, Sweden) was used for verification of aptamer immobilization and binding of protein A to immobilized aptamers. For measurements, the gold covered quartz crystals (QSX301, resonance frequency 4.95 MHz ± 50 kHz, Biolin Scientific) were cleaned as described above and mounted into the flow module QFM401. First, a baseline in BB was established, and then the chamber was flushed with a solution of 1 µM preconditioned PAA and 4 µM MCH in BB and incubated overnight while the frequency change was continuously measured. The temperature was held at 21 °C. The next day, the crystal was incubated with 1 mM MCH for 30 min and washed with BB. The modified crystals were incubated for 4 min with different concentrations of protein A (50–500 nM). Unbound protein A was washed away with BB. The relative frequency change—difference of frequency change measured in BB before and after incubation with aptamer or protein A—was used to determine the mass change by the Sauerbrey equation [52]. Thereby, most of the influences of viscosity and density of the fluids on the measurements were compensated.

### 3.4. MST Measurements

To determine the influence of ferri-/ferrocyanide on aptamer-target-binding, microscale thermophoresis (MST) measurements were performed with the Monolith NT.115 (NanoTemper Technologies GmbH, Munich, Germany) and standard capillaries. The fluorescence dye NT-547 was bound to the thiol group of PAA using the labeling kit MO-L005 Monolith™ by NanoTemper. A fluorophore-per-aptamer-ratio of 0.5 was determined by measuring the absorbance at 260 and 546 nm with a nanophotometer™ (Implen GmbH, Munich, Germany). The MST power was set to 40% and the LED power was set to 100% (no bleaching was observed). 38.5 nM labeled aptamer (77 nM in total, including the unlabeled aptamer) were incubated with 0.2 nM to 6.25 µM recombinant protein A. The analyses were performed in BBT at 25 °C. Normalization of the fluorescence signal and fitting to the Hill equation were performed using the software MO Affinity Analysis v2.1.3 (NanoTemper). To investigate if the labeling procedure or labeling site influences the aptamer affinity, additional MST measurements were conducted by commercial analysis service of 2bind GmbH (Regensburg, Germany). The same labeling procedure as described above was applied to the 3′- and 5′-end, and 30 nM labeled PAA with 0.354 nM to 11.6 µM protein A in BB with 0.05% Tween 20 were used.

### 3.5. EIS Measurements

Electrochemical impedance spectroscopy (EIS) measurements were performed using the SP-300 potentiostat/galvanostat with impedance analyzer (Bio-Logic Science Instruments SAS, Claix, France). A custom flow-through chamber (Figure 1) made of polyether-ether-ketone was designed and manufactured. Flow-through was realized by a peristaltic pump (IPC-16, Ismatec, Cole-Parmer GmbH, Wertheim, Germany) and a Teflon tube of 0.5 mm inner diameter. The fluid chamber with a volume of 100 µL was covered with glass. It consists of a three-electrode system with a working electrode cut from gold coated test slides (TA134-(Ti/Au), EMF Corporation, Ithaca, NY, USA), a Pt-black wire as counter electrode and a leak-free Ag/AgCl reference electrode (LF-1 Ø1 mm, Innovative Instruments Inc., Tampa, FL, USA). The electrochemical active area (*A_true_*) of the working electrode was determined by cyclic voltammetry in 0.5 M H_2_SO_4_ with 100 mV/s from 0.2 to 1.5 V. By integration of the reduction peak at ~800 mV, the required charge for reduction of a gold oxide monolayer was obtained (215 µC). Division by the theoretical value, 482 µC/cm^2^, calculated by [53], resulted in *A_true_* = 0.444 ± 0.039 cm^2^. The geometric surface area *A_geo_* was 0.246 cm^2^ and thus, the roughness factor *R* = *A_true_*/*A_geo_* was 1.8. The flow-through chamber and the solutions were kept in a temperature cabinet at 21 °C while the pump and impedance analyzer were positioned outside.

EIS measurements were performed with the aptamer-modified gold electrode mounted in the flow-through measurement chamber (Figure 1). Different concentrations of protein A (2–700 nM), 6 mL of each, were pumped (50 µL/s) through the chamber and incubated for 5 min while flow-through was paused. Then 6 mL FeBB was pumped (50 µL/s) through the system. Finally, the impedance was measured from 1 Hz to 200 kHz with 7 logarithmic spaced frequencies per decade. The sinusoidal alternating voltage with an amplitude of 10 mV was applied at the equilibrium potential of ferri-/ferrocyanide (*E_eq_* ~ 140 mV). The impedance measurement was repeated four times while the average of the last three cycles was used for fitting and analysis. Fitting was performed with the simplex algorithm implemented in EC-Lab® software (v11.00, Bio-Logic Science Instruments SAS, Claix, France). The same procedure was applied to control samples, protein G and L, of which 1 µM were used and bacteria cell suspensions.

## 4. Conclusions

This study provides the proof of principle for an impedimetric biosensor for the rapid detection of *S. aureus*, based on the protein A-binding aptamer. Successful co-immobilization of protein A-binding aptamers and 6-mercapto-1-hexanol on gold electrodes resulted in an average density of 1.01 ± 0.44 × 10^13^ aptamers per cm^2^. The immobilization density can be influenced by the ratio of aptamer to 6-mercapto-1-hexanol (MCH) as shown with chronocoulometry. We showed with MST measurements that ferri-/ferrocyanide, necessary as redox couple for faradaic impedance measurements, has no significant influence on the binding of the aptamer to its target. The biosensor displayed sensitive binding to protein A with a *K_D_* of 18.5 ± 1.8 nM and a LoD of 3 nM. Our results also showed the excellent selectivity of the developed sensor, with signals below LoD upon exposure to high concentrations of the functionally similar proteins G and L.

When exposed to live *S. aureus* cells, our developed aptamer-based biosensor showed a *K_D_* of 111 ± 96 CFU·mL^−1^ and a LoD of 10 CFU·mL^−1^, which is in good agreement with other reported assays or sensors. Our results also prove the high selectivity of the aptamer, distinguishing between *S. aureus* and protein A- deficient bacteria, such as *E. coli* and *S. epidermidis*.

For application in a clinical setting, an additional step for the evaluation of the different detectable *S. aureus* strains and their possible antibiotic resistance (e.g., by PCR) may have to be considered. Furthermore, the influence of different ionic strength buffers and sample matrices on the biosensor performance have to be investigated closely.

This work demonstrated that the protein A-binding aptamer can be used as recognition element in impedimetric aptasensors for successful, rapid, sensitive and selective detection of *S. aureus* in buffer. It contributes to the deeper understanding of impedimetric aptasensors and their development. We provided a fundamental base for inexpensive and robust biosensing, utilizing aptamer receptors. The advantages of using gold electrodes are their robustness, enabling regeneration and subsequent reuse of the biosensor. The simplicity of our design enables easy reproduction and the developed microfluidic system can be easily automated. Furthermore, combination with electrode patterning may enable the parallel measurement of multiple analytes when functionalized with different aptamers, in the future.

## Figures and Tables

**Table 1:** Results from the fitting of impedance data to the modified Randles circuit—SD = standard deviation, Χ/$\sqrt{N}$ = error of fitting normalized to the number of data points.

c(Protein A)/nM	R*_sol_*/Ω·cm^2^	SD	CPE/µF·s^(α-1)^	SD	α	R*_ct_*/Ω·cm^2^	SD	σ/Ω·s^−1/2^	SD	Χ/$\sqrt{N}$
7	44.4	0.2	0.362	0.005	0.95	505.3	0.5	5769	2	0.0122
24	46.2	0.1	0.375	0.004	0.94	593.3	0.5	5847	2	0.0124
70	45.4	0.1	0.359	0.004	0.95	627.9	0.5	5861	2	0.0120
240	46.1	0.1	0.341	0.004	0.95	647.3	0.5	5834	2	0.0105
700	45.3	0.1	0.361	0.004	0.95	657.2	0.5	5811	2	0.0118

**Table 2:** Apparent dissociation constant, *K_D_*, determined with different analysis methods for the protein A-binding aptamer—MST = microscale thermophoresis, SPR = surface plasmon resonance, ELONA = enzyme-linked oligonucleotide assay, EIS = electrochemical impedance spectroscopy, RIFTS = reflective interferometric Fourier transform spectroscopy.

Analysis	Aptamer	Aptamer	Protein A	*K_D_*/nM			Reference
Method		Modification					
MST	free	5′-fluorescence	free	94.7	±	64.6	[35]
MST	free	5′-fluorescence	free	115.6	±	26.9	this work
MST	free	3′-fluorescence	free	110.8	±	42.3	this work
MST	free	3′-fluorescence	free	22.4	±	5.8	this work
SPR	free	5′-fluorescence	immobilized	1920.0	±	250.0	[35]
SPR	immobilized	3′-biotin	free	287.0	±	16.2	[35]
ELONA	free	5′-biotin	immobilized	23.7	±	2.0	[36]
ELONA	free	3′-biotin	immobilized	11.3	±	1.4	[36]
EIS	immobilized	3′-thiol	free	18.5	±	1.8	this work
RIFTS	immobilized	3′-amino	free	13980.0	±	1540.0	[46]

**Table 3:** Results from the fitting of impedance data to the modified Randles circuit—SD = standard deviation, Χ/$\sqrt{N}$ = error of fitting normalized to the number of data points.

c(*S. aureus*) /CFU·mL^−1^	*R_sol _*/Ω·cm^2^	SD	CPE /µF·s^(α-1)^	SD	α	*R_ct_* /Ω·cm^2^	SD	σ /Ω·s^−1/2^	SD	Χ/$\sqrt{N}$
1E+01	14.8	0.1	1.230	0.02	0.99	459.8	0.3	310	3	0.0084
1E+02	14.8	0.1	1.232	0.02	0.99	467.8	0.3	311	3	0.0092
1E+03	14.9	0.1	1.229	0.02	0.99	474.7	0.3	312	3	0.0088
1E+04	15.0	0.1	1.224	0.02	0.99	485.7	0.3	315	3	0.0086
1E+05	15.0	0.1	1.232	0.02	0.99	492.2	0.3	312	3	0.0098
1E+08	14.9	0.1	1.231	0.02	0.99	497.9	0.3	312	3	0.0098
1E+10	14.9	0.1	1.226	0.02	0.99	491.8	0.3	317	3	0.0094

**Table 4:** Comparison of several detection methods for *Staphylococcus aureus* (SA = *S. aureus* aptamer, LoD = limit of detection).

Detection Principle	Recognition Element	Assay Time	LoD/CFU·mL^−1^	Reference
polymerase chain reaction	ssDNA	2 h	10	[47]
EIS immunosensor	anti-*S. aureus*-antibody	Not stated	10	[14]
resonance light scattering	SA 17 & SA 61 [26]	1.5 h	1	[26]
EIS	SA 43 [48]	1 h	10	[31]
fluorescent nanoparticles	SA 31 [48]	2 h	93	[24]
EIS	PA2#8[S1–58] [35]	10 min	10	this work

## Abbreviations

BB	Binding buffer
BBT	Binding buffer and 0.005% Tween 20
CFU	Colony-forming unit
*CPE*	Constant phase element
DNA	Deoxyribonucleic acid
EIS	Electrochemical impedance spectroscopy
ELONA	Enzyme-linked oligonucleotide assay
FeBB	Binding buffer and 2 mM equimolar ferri-/ferrocyanide
*h*	Hill coefficient
*K_D_*	Apparent binding constant
kDa	kiloDalton
LoD	Limit of detection
MCH	Mercaptohexanol
MDPI	Multidisciplinary Digital Publishing Institute
MRSA	Methicillin-resistant *Staphylococcus aureus*
MST	Microscale thermophoresis
ON pool	Random oligonucleotide pool
PAA	Protein A-binding Aptamer
PCR	Polymerase chain reaction
QCM	Quartz crystal microbalance
*R_ct_/*Δ*R_ct_*	Charge transfer resistance/change of charge transfer resistance
RIFTS	Reflective interferometric fourier transform spectroscopy
RMS	Rough mean square
*R_sol_*	Solution resistance
RuHex	hexaammineruthenium(III) chloride
SELEX	Systematic Evolution of Ligands by Exponential Enrichment
spA	Gene encoding protein A
SPR	Surface plasmon resonance spectroscopy
SD	Standard deviation
TSB	Tryptic soy broth
vWF	Von Willebrand factor
*W*	Warburg element
